# Malnutrition, contaminated water, and post-infectious neuropathies: lessons from the Guillain-Barré syndrome outbreak in Gaza

**DOI:** 10.1097/MS9.0000000000004396

**Published:** 2025-11-24

**Authors:** Ehtisham Haider, Tirath Patel, Christopher Hanani

**Affiliations:** a Department of Medicine, Punjab Medical College, Faisalabad, Pakistan; b Department of Surgery, Trinity Medical Sciences University School of Medicine, Saint, Vincent and the Grenadines; c Department of Neurology, Henry Ford Health System, Detroit, Michigan, USA

**Keywords:** Gaza, Guillain-Barré syndrome, malnutrition, post-infectious neuropathy, water contamination

## Abstract

The cases of malnutrition, water insecurity, and the collapse of the health system, turning into a humanitarian crisis, have been illustrated by the 2025 outbreak of Guillain-Barré syndrome (GBS) in Gaza, which is a form of treatable post-infectious neuropathy. Toward the end of August 2025, there were reports of over 90 cases and at least 10 deaths due to shortages of intravenous immunoglobulin, plasma-exchange filters, and ventilators. GBS-inducing enteric pathogens such as *Campylobacter jejuni* are likely promoted by chronic food insecurity and fecal contamination of water, but micronutrient deficiencies (e.g., thiamine) can also contribute to or exacerbate polyradiculoneuropathy. Nutritionally deficient patients cannot regenerate as well due to a reduced immune response that limits the ability to fight infections or repair nerve injuries. It is thus important to integrate nutritional surveillance and rehabilitation with the delivery of acute care that includes the administration of immunotherapy, ventilatory support, and the identification of pathogens. The Gaza experience makes it clear that an appropriate response to the outbreak must address both the urgent treatment of patients and the structural factors associated with malnutrition and water insecurity to prevent avoidable deaths and chronic disability from GBS and other post-infectious neuropathies.

*To the Editor,*

GuillainBarré syndrome (GBS) is an acute immune-mediated polyradiculoneuropathy most commonly occurring in the acute inflammatory demyelinating subtype form and is the leading cause of acute flaccid paralysis in the world. It is usually acquired following infection, primarily *Campylobacter jejuni*, influenza or other respiratory and enteric agents. Similar immune responses have also been determined to be linked to post-infectious neurological injury by other agents, such as the Zika virus and SARS-CoV-2. The disorder is usually characterized by such symptoms as symmetrical weakness, tingling, and numbness of the limbs, which are typically associated with the absence of reflexes. It starts distally and progresses to the proximal muscles, attacking the respiratory muscles and resulting in paralysis and even death, or breathing difficulties. The primary mechanism of molecular mimicry involves the development of antibodies against microbial antigens that are misdirected against peripheral nerve gangliosides, leading to demyelination and axonal damage^[1,2]^. The global incidence is estimated at 1–2 cases per 100 000 individuals per year, but rates differ among populations^[3]^. Most patients recover with timely immunotherapy and intensive care, but in areas with limited resources, patients rapidly develop respiratory failure or die^[1,2]^.

Advances over the last few years have broadened knowledge of the immunological pathophysiology of GBS, clinical patterns, and the effects of interventions such as intravenous immunoglobulin (IVIG) and plasma exchange^[4,5]^. The available evidence is mostly based on experience in well-resourced health systems. Malnutrition and unsafe water may affect both the susceptibility and disease outcomes in areas with less developed health infrastructure, although this is not well researched.

Malnutrition undermines immune response and mucosal defense making a person more vulnerable to infection and slow nerve repair^[6]^. Deficiencies such as lack of thiamine may also mimic or worsen neuropathic syndromes, making diagnosis difficult^[7]^. In parallel, the collapse of sanitation and water infrastructure increases exposure to pathogens such as *Campylobacter*, one of the clearest infectious triggers of GBS^[8]^. These conditions together raise both the likelihood of developing the disease and its clinical severity once it occurs.

This pattern has become evident in Gaza, where, by late August 2025, more than 90 confirmed cases and at least 10 deaths had been reported^[9]^. Hospitals continue to struggle with shortages of IVIG, plasma exchange filters, and ventilators^[10]^. Food insecurity and unsafe drinking water are leaving many people malnourished and immunocompromised^[11,12]^. These overlapping crises have created an environment in which enteric infections spread more easily, while the body’s immune defenses are already weakened. Clinicians have reported rapid deterioration in affected patients, reflecting how malnutrition, infection, and limited access to care combine to worsen outcomes^[9,10]^.

Effective action should tackle not only the immediate problems but also the factors that drive them. Surveillance for GBS and acute flaccid paralysis should include pathogen identification and nutritional assessment. Nutritional programs providing therapeutic feeding and micronutrient support should be integrated into outbreak control. Providing access to IVIG, ventilatory support, and necessary supplies is essential. In the longer term, studying the interaction between infection and malnutrition in crisis settings can guide planning and help prevent neurological complications in future emergencies. Clinicians and public health authorities must therefore advocate for emergency neurology preparedness that accounts for nutritional and infrastructural determinants of infection-related neuropathies.

The situation in Gaza shows how the integration of malnutrition, contaminated water, and a failing health system can transform a treatable post-infectious neuropathy into a significant public health emergency. Addressing immediate treatment needs while confronting these structural determinants remains essential to prevent avoidable deaths and long-term disability from GBS in humanitarian contexts.

This manuscript is made compliant with the TITAN checklist to ensure transparency in the reporting of Artificial Intelligence^[13]^.

## Data Availability

No new dataset was generated during the study.

## Research registration unique identifying number (UIN)

Not required because the study is a letter to the editor.

## References

[1] Hunter J English WA Wiegers EJA. Guillain-barré syndrome. BJA Educ 2025;25:309–16. 40693120 10.1016/j.bjae.2025.05.001 PMC12276597

[2] Hughes RA. Guillain-Barré syndrome: history, pathogenesis, treatment, and future directions. Eur J Neurol 2024;31. doi:10.1111/ene.16346 PMC11464409 38752584

[3] Jeong YD Park S Lee S. Global burden of vaccine-associated guillain-barré syndrome over 170 countries from 1967 to 2023. Sci Rep 2024;14. doi:10.1038/s41598-024-74729-2 PMC11490553 39427003

[4] Review for expanding our understanding of guillain–barré syndrome: recent advances and clinical implications. Eur J Immunol 2024;11:e2250336. 10.1002/eji.202250336 39188201

[5] Gorson KC. Evolving understanding of guillain-barré syndrome pathophysiology and the central role of the classical complement pathway in axonal injury. Front Neurol 2025;16:1572949. 10.3389/fneur.2025.1572949 PMC12117664 40438570

[6] Morales F Montserrat-de la Paz S Leon MJ. Effects of malnutrition on the immune system and infection and the role of nutritional strategies regarding improvements in children’s health status: a literature review. Nutrients 2023;16:1. 38201831 10.3390/nu16010001 PMC10780435

[7] Benameur K, and Clarke K. Thiamine deficiency masquerading as guillain-barré syndrome. Cureus 2025;17:e78310. 10.7759/cureus.78310 PMC11872742 40034881

[8] Poropatich KO Walker CL Black RE. Quantifying the association between campylobacter infection and guillain-barré syndrome: a systematic review. J Health Popul Nutr 2010;28:545–52. 21261199 10.3329/jhpn.v28i6.6602 PMC2995022

[9] Gaza. Hospitals “at near-total collapse,” staff overwhelmed by the injured. UN News 2025. Accessed 1 October 2025. https://news.un.org/en/story/2025/08/1165592

[10] Poidevin OL Critical medical supplies run out as cases of rare syndrome rise in Gaza, WHO says. Reuters. 2025 Aug 29. https://www.reuters.com/business/healthcare-pharmaceuticals/critical-medical-supplies-run-out-cases-rare-syndrome-rise-gaza-who-says-2025-08-29/

[11] World Health Organization. Famine confirmed for first time in Gaza. WHO. 2025. Accessed 1 October 2025. https://www.who.int/news/item/22-08-2025-famine-confirmed-for-first-time-in-gaza

[12] UNICEF. WASH: water, sanitation and hygiene in the State of Palestine: situation report. UNICEF. 2025. Accessed 1 October 2025. https://www.unicef.org/sop/topics/water-sanitation-and-hygiene

[13] Agha RA Mathew G Rashid R. TITAN Group. Transparency in the reporting of Artificial Intelligence– the TITAN guideline. Prem J Sci 2025;10:100082.

